# Enactivism and neonatal imitation: conceptual and empirical considerations and clarifications

**DOI:** 10.3389/fpsyg.2014.00967

**Published:** 2014-09-02

**Authors:** Paul Lodder, Mark Rotteveel, Michiel van Elk

**Affiliations:** Department of Psychology, University of AmsterdamAmsterdam, Netherlands

**Keywords:** enactivism, neonatal imitation, intersubjectivity, action understanding, social cognition

## Abstract

Recently within social cognition it has been argued that understanding others is primarily characterized by dynamic and second person interactive processes, rather than by taking a third person observational stance. Within this enactivist view of intersubjective understanding, researchers differ in their claims regarding the innateness of such processes. Here we proposed to distinguish nativist enactivists—who argue that studies on neonatal imitation support the view that infants already have a non-mentalistic embodied form of intersubjective understanding present at birth—from empiricist enactivists, who claim that those intersubjective processes are learned through social interaction. In this article, we critically examine the empirical studies on neonate imitation and conclude that the available evidence is at least mixed for most types of specific gesture imitations. In the end, only the tongue protrusion imitation appears to be consistent across different studies. If neonates imitate only one single gesture, then a more parsimonious explanation for the tongue protrusion effect could be put forward. Consequently, the nativist enactivist claim that understanding others depends on second person interactive processes already present at birth seems no longer plausible. Although other strands of evidence provide converging evidence for the importance of intersubjective processes in adult social cognition, the available evidence on neonatal imitation calls for a more careful view on the innateness of such processes and suggests that this way of interacting needs to be learned over time. Therefore the available empirical evidence on neonate imitation is in our view compatible with the empiricist enactivist position, but not with the nativist enactivist position.

## 1. Introduction

Humans are social in nature. Almost everything we do involves interacting with other human beings. An important prerequisite for social interaction is the understanding of others ^1^. Take for instance a game with three people in which person A reads a message and has to transfer it to person B, who, after receiving the message has to transfer it to person C. The difficulty in this game, however, is that person A and C are not allowed to interact directly and all attendants are not allowed to use spoken language. Therefore they have to transmit the message by only using weird sounds and gestures instead. Often the receiver of the message imitates the gestures and sounds of the transmitter in order to better understand the transmission. In the end, the original message is compared to person C's interpretation of the message received from person B. Occasionally, person C's interpretation differs considerably from the original message, but surprisingly often the interpretation lies close to the original message. This example not only illustrates that human interaction requires us to understand each other's actions, but it also shows that we are pretty good at it, even in complex situations where we cannot use all available channels of communication. But how exactly are we able to understand actions of other people?

Within the field of social cognition, there are two dominant theoretical approaches that explain our ability to understand other human beings form a cognitivist perspective. According to *Theory theory* (TT), we understand others by theorizing about their minds (Leslie, 1987; Gopnik and Wellman, 1994). On this account, the understanding of other minds relies on taking a theoretical stance and postulating the existence of mental states in others that can help us to explain their behavior. *Simulation theory* (ST), on the other hand, posits—broadly speaking—that we use our own experiences as an internal model for understanding others (Gordon, 1986; Goldman, 2002). We simulate thoughts and/or feelings that we would experience if we were in the very same situation the other person is in. TT and ST agree about the fact that we explain and predict other's behavior using mental state attributions by taking a third person observational stance. Because both theories use internal representations to explain how human beings understand others, they can be viewed as representational theories. The nature of these representations, however, differs between the two theories and they therefore disagree clearly with regard to the processes that let us understand others. TT claims that understanding others can be accomplished by using abstract theories about other minds, while ST claims that representations are based on sensorimotor experiences instead and involve simulating others' thoughts and actions.

Recently, it has been argued that understanding others is not primarily characterized by taking such a third person stance involving representations of other's actions, but instead by a second person stance involving dynamic and interactive processes (Zahavi, 2001; Gallagher, 2005; Gallagher and Hutto, 2008; Fuchs and De Jaegher, 2009). This enactivist position proposes that the environment as well as an agent's body play an important role in shaping our cognition. According to enactivists, cognition is a sense making process, emerging from a dynamic interaction between agents and the environment in which they are embedded (de Bruin and Kästner, 2012). Enactivist theories are for instance supported by studies on motoric development in children, showing that their stepping behavior does not result from a cognitive programme present in the child, but instead the behavior self-organizes in a dynamic interaction between a child's spontaneous limb movements and a changing environment (Galloway and Thelen, 2004; Gershkoff-Stowe and Thelen, 2004). The enactivist proposal differs from both third person perspectives on social cognition (Theory theory and Simulation theory) in that the latter two use internal representations to explain our understanding of others, while enactivism is strongly anti-representational (Chemero, 2009). While this anti-representationalism is an essential characteristic of enactivism in general, enactivists still argue about the origins of the intersubjective processes we use to understand others. Some argue that these processes are innate and therefore already present at birth (Gallagher, 2001, 2005; Gallagher and Hutto, 2008; Fuchs, 2009), a position coined *nativist* enactivism. *Empiricist* enactivists, on the other hand, claim that these intersubjective processes are not innate, but develop as a result of interpersonal interaction (Di Paolo and De Jaegher, 2012; Froese et al., 2012)^2^.

Nativist enactivism does not necessarily imply a rejection of the empiricist notion that infants develop intersubjective understanding through learning. A nativist enactivist could view the processes underlying social cognition as primarily innate, while allowing experience to play a secondary role. Consequently, learning could still influence human cognition as a trigger of innately determined intersubjective processes (Gallagher, 2005). A much more stronger nativist claim would be to deny any influence of learning on human understanding whatsoever. However, such *final state* nativism (Meltzoff, 2002) is rare within enactivism, because it is incompatible with the central enactivist tenet that social cognition is shaped by experience in a dynamic interaction between an agent's body and the environment. To our knowledge, most nativist enactivist therefore still allow learning to play a role in shaping cognition (Zahavi, 2001; Gallagher and Hutto, 2008; Fuchs, 2009).

The nativist enactivist view on intersubjective understanding is supported by studies on intentionality detection (Meltzoff, 1995), eye direction detection (Baron-Cohen, 1997), and neonatal imitation (Meltzoff and Moore, 1977), suggesting that very young infants already have a non-mentalistic and embodied form of intersubjective understanding (Gallagher, 2008). Of those three strands of research, the studies on neonatal imitation are most important to the nativist enactivist view because they could imply that a basic form of intersubjective understanding is already present at birth and does therefore not depend on any learning—as, for instance, assumed by empiricist enactivists. More specifically, studies on neonatal imitation imply that a basic form of intersubjective understanding is reflected in the infant's ability to automatically and dynamically respond to observed actions, by producing a similar gesture, suggesting an important role for an innate body schema guiding interaction with the world (Gallagher, 2005). Recent reviews on neonatal imitation literature, however, questioned the generality of neonatal imitation and proposed alternatively more parsimonious theories to explain these findings (Anisfeld, 1991; Jones, 2009; Ray and Heyes, 2011).

In contrast, the empiricist view on enactivism puts more emphasis on the importance of sensorimotor and social learning for intersubjective understanding (Di Paolo and De Jaegher, 2012; Froese et al., 2012). In support of this account it is for instance pointed out that imitation in infants is experience-dependent and possibly mediated by the sensorimotor configuration of the so-called mirror neuron system (MNS). Furthermore, it is argued that rather than being equipped with an innate body schema, infants gradually acquire an implicit sense of their body through visuomotor and visuo-tactile experience (Zmyj et al., 2011).

In the present paper we investigate whether the available empirical evidence for neonatal imitation poses a potential problem for the validity of the nativist enactivist claim that understanding others depends on second person interactive processes that are already present at birth. If neonates can imitate only one single gesture, then a more parsimonious explanation could be put forward. Therefore, we will investigate the scope of neonatal imitation, because the nativist enactivist theories rely on the generality of this phenomenon (Heyes, 2001). First, we will clarify the basic concepts and theories about imitation, followed by a short review of the classic neonate imitation experiments by Meltzoff and Moore (1977, 1983a, 1989, 1994). After that we will focus on some contradictory findings, followed by an examination of two systematic reviews (Anisfeld, 1991; Ray and Heyes, 2011). Lastly, we will wrap these findings up and consider their implications for the enactivist approach on intersubjective understanding.

## 2. Imitation

One of the milestones in parent-child interaction is the moment a newly born for the first time imitates the parent. Examples of such mimicking behavior are the imitation of observed head movements, facial gestures, or even rudimentary speech. Imitations are not confined to human beings: researchers demonstrated that birds and non-human primates are also able to imitate, even at a neonatal age (Carpenter and Tomasello, 1995; Custance et al., 1995, 1999; Akins and Zentall, 1996, 1998; Ferrari et al., 2006; Myowa-Yamakoshi, 2006; Bard, 2007).

### 2.1. Definition

A key issue within imitation debates is how genuine imitation is defined, hence how the construct of imitation is validated in different empirical studies. All definitions of imitation have in common that they entail an observer copying a body (part) movement of a model (Heyes, 2001). In other words, an observer receives visual information about an observed body movement and uses this information to perform a similar movement in response. Note that we exclude those situations in which the model's movement and the imitator's movement spontaneously co-occur. We also exclude any act to be of imitative nature when it is caused by something else than the model and its behavior (Anisfeld, 1991).

Further, it is important to distinguish imitation from both emulation (Tomasello, 1996) and spatial compatibility (Brass et al., 2001). Emulation—like imitation—concerns a person copying an action from a model, but the performed action is only similar to the model's action in terms of the goal and not in terms of the movements that lead to that goal. For instance, you might water the plants with a watering can, while I might achieve the same goal by using a watering hose. In that case, the goal of the action is the same, whereas the movements differ and this is considered an instance of emulation rather than imitation. Thus, a prerequisite for genuine imitation is a match between the observed and the performed movements. Spatial compatibility—like imitation—involves a similarity between the relative position of the action of an imitator and a model, but with spatial compatibility the action's target is not necessarily similar. For instance, if a person standing opposite to you asks you to raise your right hand and he raises his own right hand at the same time, due to spatial compatibility you will be more likely to raise your own left hand instead. Emulation as well as imitation can also be used in order to understand the actions of others (Takahashi et al., 2010). That is, being able to imitate another person's actions implies the ability to respond to the other's movements in a way that is socially and communicatively effective.

### 2.2. Current debates in imitation research

Within the field of imitation research, different debates regarding the onset, the underlying mechanisms and automaticity of imitation can be discerned. Although most scientists agree that human infants *are* able to imitate at some age, probably an equal number of scholars disagree about the *exact* age at which infants become able to show imitation. Numerous studies indicate that in their second year of life infants are able to imitate other people (Piaget, 1946; Meltzoff, 1995; Carpenter et al., 1998; Nadel and Butterworth, 1999). Yet, when it comes to imitation at a *neonatal* age, the results are still contradictory (Meltzoff and Moore, 1977, 1983a; Koepke et al., 1983; McKenzie and Over, 1983).

The second dispute concerns the underlying mechanisms of imitation and whether these differ between neonatal and older infants or even adults. In a way this debate mirrors also the nature-nurture debate, because the issue is here whether imitation is innate or depends on learning. If newly born infants can imitate, then this underlines the existence of an innate mechanism underlying imitation (e.g., an automatic coupling of observed actions to one's own behavioral repertoire). When neonatal imitation proves not to be genuine, on the other hand, and is not comparable to imitation seen in older infants, then this might indicate dependency of additional learning such as learning to couple observed actions to one's own behavioral repertoire (Anisfeld, 1991; Gallagher, 2001, 2005; Ray and Heyes, 2011)^3^.

Related to this debate is the third dispute to what extent imitation in adults can be viewed as automatic (Heyes, 2011). Studies on automatic imitation in adults suggest that the mirror neuron system (MNS) provides a direct connection between the perception of action and the production of action (Kilner et al., 2003; Press et al., 2005; Longo et al., 2008; van Schie et al., 2008). This involvement of the mirror neuron system (MNS) in imitation might imply that the system has evolved as a specialized mechanism for our intersubjective understanding (Rizzolatti et al., 2001; Gallese et al., 2004). On the other hand, it has been argued that the mirror neuron system is not an innate mechanism but relies on sensorimotor learning and accordingly develops through experience (Ray and Heyes, 2011). Thus, a similar discussion regarding innateness and automaticity vs. the role of experience and learning can be observed in studies on infant imitation and the development of the MNS.

### 2.3. Functional and cognitive mechanisms

An important functional mechanism underlying imitation concerns the mapping from observed movements to one's own body. More specifically, this *correspondence problem* entails that when imitating someone, the imitator needs to know which observed body parts map onto his or her own body parts. In other words: it needs to be specified how visual information is translated into a corresponding motor act. If you see someone move their hand then you need to know that their hand looks similar to your own hand and that you are able to perform the same movement with your hand. This process becomes much more complicated when it involves the observation of body parts that are difficult to observe on your body, such as for instance your tongue. In order to solve the correspondence problem, cognitivist theories propose that infants imitate an observed movement by using an internal representation of the observed body part. Infants then associate this observation with a motor act by mentally matching this representation with proprioceptive information of their own body parts (Schaal, 1999; Heyes, 2002; Spaulding, 2010). Enactivist theories, on the other hand, propose that cognitive internal representations are not required to explain imitation. Enactivists propose that we understand other people primarily by directly responding to other people's behavior in a dynamic interaction between the environment and our own perceptual experiences.

Within enactivism, two different explanations of imitation can be distinguished. First, *nativist enactivists* claim that an innate body schema enables children to directly map observed movements (e.g., facial gestures) on their own movement repertoire. A body schema is defined as a system of sensorimotor processes that constantly regulates posture and movement—processes that function without reflective awareness or the necessity of perceptual monitoring (Gallagher, 2005). Such an innate body schema is biologically based and already present in the pre-natal stage (i.e., in the womb), where the child can already explore his own body through touch and proprioception (Butterworth, 1992; Gallagher, 2008). Nativist enactivist theorists claim that we understand other people primarily because of our *innate* capability to directly respond to other people's behavior involving a dynamic interaction between the environment and our own perceptual experiences and body schema (Gallagher, 2008). Support for the innateness of this process relies heavily on experimental studies showing that neonates already have a basic form of intersubjective understanding. If neonates have the capacity to dynamically interact with the environment by directly matching their proprioceptive experience with other people's behavior, then the basic mechanisms that adults use to understand others are already present at birth and do therefore not need to be learned. According to one nativist enactivist, the “studies on newborn imitation suggest that there is at least a primitive body schema from the very beginning. This would be a schema sufficiently developed at birth to account for the ability to move one's body in appropriate ways in response to environmental, and especially interpersonal, stimuli” (Gallagher, 2005). Similarly, according to Gallagher and Meltzoff (1996) the evidence on neonate imitation “suggests that there exists an innate system that accounts for the possibilities of early infant imitation.” This line of reasoning indicates clearly that studies on neonatal imitation are of high importance to the nativist enactivist claim.

Nativist enactivists often refer to one particular set of studies on neonate imitation published by Meltzoff and colleagues (Meltzoff and Moore, 1977, 1983a, 1989, 1994). They use these studies to support the notion that the basic intersubjective mechanisms underlying adult social cognition are already present in neonatal infants. For instance, according to Fuchs (2009), the studies by Meltzoff and Moore show “that the capacity of imitation in human infants is essential for understanding others. From birth on, infants possess interpersonal body schemas for spontaneous facial imitation and emotional resonance. They experience the other's body as similar to their own, and thus, they also transpose the seen facial expressions and gestures of others into their own feelings. These schemas underlie the development of more sophisticated empathic abilities in the course of early interactions.” In a similar vein, Gallagher and Hutto (2008) claim that the Meltzoff and Moore studies imply that “an intermodal tie between a proprioceptive sense of one's body and the face that one sees is already functioning at birth.” In other words, these studies “confirm the existence of an innate body representation,” allowing infants to “imitate some simple movements like protrusion of tongue” (De Vignemont, 2003).

The neonate imitation studies underlining the nativist enactivist claim (Meltzoff and Moore, 1977, 1983a, 1989, 1994) are, however, only a selective sample of all the studies conducted using the imitation paradigm; most other studies show at least contradictory results regarding the capability of genuine imitation in neonates. To our knowledge, most nativist enactivists do not refer to these contradictory findings (Gallagher, 2000, 2001, 2005, 2008, 2011; Zahavi, 2001; Gallagher and Hutto, 2008; Fuchs, 2009). Furthermore, the nativist enactivist's claim that neonates already have a basic form of intersubjective understanding relies heavily on experiments showing that neonates cannot only imitate one specific gesture but that they can imitate different kinds of social gestures. This generality of neonatal imitation is important to nativist enactivists: if imitation is an innate mechanism used for intersubjective understanding, then one would expect that this imitative mechanism is not limited to only one specific type of gesture. Reacting to only one specific gesture would probably indicate that neonates do not understand action in social situations but only imitate one particular gesture as a result of other, more unspecific biological, reflex-like, or learned mechanisms (Anisfeld, 1991, 1996; Heyes, 2001; Di Paolo and De Jaegher, 2012). As a consequence the nativist enactivist claim regarding the innateness and automaticity of imitation and action understanding would no longer be valid.

*Empiricist enactivists*, on the other hand, claim that the processes underlying imitation are dynamically learned during social interaction (Di Paolo and De Jaegher, 2012; Froese et al., 2012; Froese and Leavens, 2014). These views are substantiated by studies showing that the mirror system is continuously shaped through sensorimotor learning and therefore highly adaptive. This high plasticity of the mirror system enables the mechanisms underlying imitation to be constantly adjusted during interpersonal interaction (Catmur et al., 2007, 2009). We consider the distinction between nativist- and empiricist enactivism to be important, because it highlights the opposing views within enactivism regarding the origins of intersubjective understanding in humans. The studies on neonate imitation are important within this debate, because they are used to support the nativist enactivist view that those intersubjective processes are already present at birth. Although most empiricist enactivists are well aware of the conflicting evidence on neonate imitation (Di Paolo and De Jaegher, 2012; Froese et al., 2012; Froese and Leavens, 2014), some nativist enactivists clearly use the studies on neonate imitation as if they are an indisputable phenomenon (Gallagher, 2005; Gallagher and Hutto, 2008; Fuchs, 2009). Therefore, in the following paragraphs we will critically examine the studies on neonate imitation and consider the implications of these studies for both the nativist- and empiricist enactivist view on intersubjective understanding.

## 3. Experimental evidence on neonatal imitation

Studies on neonatal imitation are important within the imitation debate because they could imply that a basic form of intersubjective understanding is already present at birth and does therefore not need to be learned. The phenomenon of neonate imitation was already widely reported in the pre-experimental literature (Stern and Barwell, 1924; McDougall, 1926; Piaget, 1946), but the novelty of the Meltzoff and Moore (1977) studies was that they were the first to investigate neonate imitation in an experimental and systematic fashion, by studying infants in a hospital lab.

### 3.1. Meltzoff and moore's seminal studies

In one experiment, Meltzoff and Moore (1977) asked a model to present three different facial gestures to 12–17 days old infants. The model first presented each infant for 90 s with a neutral and passive face, which served as a baseline measure with which the imitation effect would be compared. Subsequently, the model showed the infants four times in a 15 s period randomly one of the three facial gestures (tongue protrusion, mouth opening, or lip protrusion). This was followed by a 20 s period during which the infants were allowed to respond. For all infants, responses to the model's gestures were videotaped. Afterwards and for each trial, six independent graduate students who were blind to the model's specific gestures, watched the video and ranked the facial gestures from being most to least likely imitated by the infant. For instance, a possible ranking of imitative responses for a modeled tongue protrusion could be (1) tongue protrusion; (2) mouth opening; (3) lip protrusion. It turned out that for each modeled gesture infants were significantly more likely to perform specifically that gesture, compared to no gesture or other gestures. This finding conforms the definition that imitation involves a non-random copy of an observed body (part) movement of a model caused by nothing else than the mere observation of the model itself.

One limitation of this study, however, is that the researchers did not exclude the possibility of an experimenter bias. That is, during the experiment, neonates were often not paying attention to the model, because they were spitting or choking. To overcome this problem, the model sometimes repeated the facial gesture to make sure the gestured was attended by the neonate. Consequently, this solution might have led the model to repeat the gesture until a neonatal reaction randomly coincided with the model's demonstrated gesture. To overcome this considerable problem, Meltzoff and Moore designed another experiment (Meltzoff and Moore, 1983a) in which they used a fixed duration for each presented gesture. Neonates in this experiment were even younger than those in the previous experiment: their ages ranged from 42 min to 71 h. Again, neonates imitated the model's tongue protrusion and mouth openings consistently. The effect of lip protrusion on imitation, however, failed this time to reach the required level of statistical significance.

An alternative account of this neonate imitation effect entails an innate and evolutionary relatively old release mechanism involved in promoting the neonate's chances of survival (Jacobson, 1979; Bjorklund, 1987). Mouth openings and tongue protrusions, could for instance just be a reflex toward a suckable object, such as a mother's nipple. Consequently, neonate responses in the gesture imitation paradigm could thus be caused by their mere perception of the model's tongue as a suckable object, independent of any genuine imitation. According to the innate release mechanism account, the observed link between a model's tongue protrusion and the neonate's tongue protrusion could be merely coincidental and uninformative regarding genuine imitation.

However, Meltzoff and Moore (1994) propose that if this innate release mechanism plays a role in neonate imitation, then the neonate's response to a suckable stimulus should occur shortly after the perception of that stimulus and not after a delay. To rule out the innate release account, they conducted an experiment similar to their previous experiments, but now with an additional condition in which the neonate's response was delayed by 24 h: the model randomly demonstrated a gesture and after 24 h, the neonates saw the same model again, but now only with a passive face. First, Meltzoff and Moore replicated their previous findings that neonates systematically imitated the model's tongue protrusion and mouth openings if they were allowed to respond directly after the model presented the gesture. Furthermore, after the 24 h delay, neonates showed significantly more tongue protrusions than other gestures, if the model had demonstrated a tongue protrusion 24 earlier. Interestingly, this effect was not found for other gestures. This finding is interpreted as reflecting a specific effect of imitation, in which the observed action is imitated after a delay and can therefore not be explained by being a reflex due to an innate release mechanism^4^.

Several other studies found results very similar to those of Meltzoff and Moore (Jacobson, 1979; Field et al., 1983; Meltzoff and Moore, 1983b; Fontaine, 1984; Kugiumutzakis, 1985; Abravanel and DeYong, 1991), but an even more extensive number of studies failed to replicate these initial neonate imitation effects (Anisfeld et al., 1979; Hayes and Watson, 1981; Koepke et al., 1983; McKenzie and Over, 1983; Neuberger et al., 1983; Abravanel and Sigafoos, 1984; Fontaine, 1984; Lewis and Sullivan, 1985; Heimann et al., 1989). To clarify and explain these mixed results, several reviews on neonatal imitation have been published that will be discussed in the next section.

### 3.2. Reviews of neonatal imitation

One review analyzed 26 experiments on neonatal imitation that together combined 15 different gestures in a total number of 76 gesture conditions (Anisfeld, 1996). Tongue protrusion and mouth opening were the most commonly studied gestures, accounting for 23 and 16 gesture conditions, respectively. Anisfeld counted for each experiment whether or not an effect was found in a particular gesture condition. He defined an effect as present when the neonates showed significantly more correct imitations in the gesture condition than in the neutral comparison condition. Finally, he required an effect to be significant on a two tailed test, with a *p*-value smaller than 0.05.

In total, an effect was present in 28 of the 76 gesture conditions (37%). It turned out that an effect was present in 12 of the 23 tongue protrusion conditions (52%), 3 of the 16 mouth opening conditions (19%), and 13 of the 37 remaining gesture conditions (35%). Tongue protrusion appears thus to be stronger than the other gesture effects in this review. However, still 48% of the tongue protrusion conditions did not show an effect at all. For all 11 tongue protrusion conditions that did not have a significant effect, the duration of the gesture demonstration turned out to be less than 40 s. Conversely, conditions in which the tongue protrusions were demonstrated for more than 60 s all did show a significant effect. Anisfeld (1991) concludes therefore that a neonate imitation effect is present only for the tongue protrusion gesture and only under conditions of longer gesture presentation.

Based on the review, Anisfeld (1996) argues further that if neonate imitation would have been a general phenomenon, then neonates that showed a strong tongue protrusion effect should also more strongly imitate other studied facial gestures. In other words, if genuine neonate imitation is present, then a positive correlation should show up between different gesture imitations. This was, however, not the case for the 76 reviewed gesture conditions (Anisfeld, 1996).

Anisfeld investigated additionally also the frequency of tongue protrusions and mouth openings per minute after modeled tongue protrusions, mouth openings, or passive faces. He found that the frequency of neonatal tongue protrusions was significantly higher after a modeled tongue protrusion than after modeled mouth openings or passive faces. This effect was not found for the mouth openings: the frequency of mouth opening responses did not significantly differ when either tongue protrusions, mouth openings or passive faces were modeled. This does not necessarily mean however that no genuine imitation of mouth openings was present. It could also mean that statistical power was simply too low. That is, Anisfeld analyzed a total of 12 mouth opening studies. The power to find a medium effect (*d* = 0.50), given an alpha of 0.05 and a sample size of 12, equals 0.35, which is quite low indeed (Cohen, 1977).

Furthermore, because Anisfeld used data from different studies in his two-sided *t*-test, the observations of the neonates are nested within the different studies, making it likely that specific study characteristics influence the neonate imitation effects excessively (Hox, 2002). In his analysis, Anisfeld also made use of aggregated data by looking at the mean frequencies of neonatal gesture responses, thereby ignoring individual variation in gesture responses. In fact, even more variation is ignored because the data actually conforms to a multilevel structure with four levels: gestures nested within neonates, nested within experiments, nested within studies. When a multilevel analysis had been adopted instead, then this unsystematic variation would have been addressed more appropriately. By not taking this variation into account, chances of making a type I error are dramatically increased (Stevens, 2009; Hox, 2010), which makes it also more likely that the tongue protrusion imitation is over-estimated or even is itself a false positive.

These latter statistical considerations make it difficult to conclude clearly about the presence or absence of neonatal imitation based on the analysis of the tongue protrusion and mouth opening frequencies. This leaves us then with Anisfeld's counts of the significant gesture effects showing significance for only 52% (12/23) of the tongue protrusion conditions and 37% (28/76) of the gesture conditions in general. However, this analysis simplifies and reduces quantitative information by dichotomizing the data into either an effect or no effect. The strength of an effect or the amplitude is thereby completely ignored, as well as the variation of the data within each separate study. Therefore, we cannot draw any strong conclusions about the strength of the genuine neonate imitation effects for each gesture. This would only be possible if we conduct a meta-analysis, but most of the reviewed studies did not even report standard deviations, which makes it impossible to conduct a proper meta-analysis in the first place (Tabachnick et al., 2001)^5^.

A more recent review corroborates the findings of Anisfeld (1996). Ray and Heyes (2011) reviewed 37 experiments on neonatal imitation, comprising a total of 17 different gestures. It turned out that eight of those gestures did not provide support for the existence of genuine neonatal imitation. Eight of the remaining nine gestures showed mixed results, but the authors explained these findings either as peculiar scoring criteria, or by being a side-effect of the tongue protrusion gesture. Peculiar scoring criteria include for instance the categorization of each imitation as either present or absent, rather than calculating response frequencies. Furthermore, gestures that include mouth movements such as mouth openings can be viewed as a side-effect of an imitated tongue protrusion. Despite these limitations, but in line with the results of Anisfeld (1996), the only gesture that did reliably show positive results was the tongue protrusion (Ray and Heyes, 2011).

Because the reviews described in this paper lack proper meta-analytic techniques, a compelling meta-analysis seems to be required to settle the question whether neonatal imitation really exists. Additionally, one venue for further empirical exploration of this matter could be to find out which factors may moderate the neonate imitation effects (e.g., differences in parental style and personality characteristics, attractiveness of the experimenter's face, delay that is used in the experiment etc.). Moderating factors might explain the huge discrepancy in the experimental findings that have been reported thus far. A proper meta-analysis will not only overcome the statistical problems of the systematic review by Anisfeld (1996), but it can also be used as a tool to discover factors moderating the neonate imitation effects.

## 4. Discussion

The studies reviewed above indicate that there is no convincing evidence for the existence of neonatal imitation of different social gestures. Both reviews conclude that only the tongue protrusion gesture shows a reliable imitation effect (Anisfeld, 1991; Ray and Heyes, 2011). However, these reviews suffer from a number of statistical flaws that make it difficult to interpret their results decisively in this matter. Leaving this aside, the Anisfeld (1991) review points out that 63% of the investigated imitation conditions failed to show any effect, which indicates at least that the available evidence does not favor neonatal imitation in general. And although the strongest imitation effect appears to be found with tongue protrusion gestures, still 48% of those experiments fail to find an effect. Thus, it can be concluded that neonate imitation is far from a well-established scientific phenomenon. It seems misleading therefore to present genuine neonate imitation as a robust finding (as for instance in Gallagher, 2005, and see Gallagher, 2000, 2001, 2005, 2008, 2011; Zahavi, 2001; Gallagher and Hutto, 2008; Fuchs, 2009; Varga and Gallagher, 2012).

### 4.1. Alternative accounts of the empirical evidence on neonatal imitation

If neonates are really capable of genuine imitation, then nativist enactivists need to explain why the experimental evidence is so contradictory and why it seems to indicate that genuine neonate imitation—if it exists at all—is only restricted to tongue protrusions. If neonate imitation is not a general phenomenon, then it is more parsimonious to explain tongue protrusions, for instance, by an underlying innate release mechanism (Anisfeld, 1996). According to this interpretation, a modeled tongue protrusion resembles an approaching nipple, thereby triggering an innate sucking reflex in the neonate. This interpretation cannot explain, however, the finding of delayed tongue protrusions observed in one of Meltzoff and Moore's experiments (Meltzoff and Moore, 1994), because the innate release mechanism requires the reflex to happen directly after the observed tongue protrusion.

An even more parsimonious explanation that also does *not* contradict Meltzoff and Moore's delayed response finding (Meltzoff and Moore, 1994), proposes that tongue protrusions reflect a tendency to explore the world (Jones, 2009). One study showed, for instance, that neonates do not only stick out their tongue in reaction to a tongue or nipple-like objects, but also to a human face or inanimate objects such as bright lights or music (Jones, 1996a). Consequently, this theory explains the delayed tongue protrusion as oral exploratory behavior in reaction to non-specific visual stimuli – in this case the mere perception of the person who modeled the tongue protrusion 1 day earlier. This implies that to a neonate, modeled tongue protrusions are just a specific example of a wide range of stimuli that can arouse the neonate's interest to explore the world. Additionally, a longitudinal study indicates that tongue protrusions decrease as soon as infants become able to grasp objects (Jones, 1996b). Therefore, according to Jones, the tongue protrusion effect can be more parsimoniously explained as an innate reflex that enables neonates to start exploring the world until other modes of exploration become possible. The finding that tongue protrusions are not only directed at humans but also at inanimate objects like bright lights, suggests that tongue protrusions do not necessarily have a communicative or social function. However, if the tongue protrusions directed at humans are of a different kind than those directed at inanimate objects, then a social function might still be possible alongside the gesture's explorative features as proposed by Jones (2009).

Both alternative explanations described above propose that neonate imitation is caused by an innate, reflex-like mechanism and does not reflect genuine imitation as defined before. Although both explanations can explain the origin of the tongue protrusion imitation in neonates, they cannot account for instances of infant or adult imitation that are more complex, such as intentional imitation. This naturally raises the question of how and by what mechanisms human beings are able to develop the capacity to imitate. Recently, a new model has been proposed that explains imitation as a process that is learned through sensorimotor experience, rather than a purely innate biological mechanism (Heyes and Ray, 2000; Ray and Heyes, 2011). This *associative sequence learning* (ASL) model claims that associations between motor representations and sensory representations of an action are formed through experience via associative learning (Schultz and Dickinson, 2000). These associations can be formed not only through direct self-observation, but also by observing oneself through a mirror or by observing someone else imitating your actions. In this way, the ASL model is able to explain how infants learn to imitate—even the imitation of actions that cannot be directly observed by the actor, such as for instance facial expressions.

Various studies support this notion that genuine imitation is acquired through learning rather than being innate. First, evidence from neuroimaging studies indicates that sensorimotor experiences can influence the mirror neuron system (Calvo-Merino et al., 2005, 2006). For instance, people who are expert dancers show more activity in their mirror neuron system when observing other people perform “their” dance, than when they observe a dance they do not master. This difference in mirror neuron system activity might imply that sensorimotor learning influences the development of the mirror neuron system. This connection between action experience and action observation is also found in young children. Sommerville et al. (2005) showed that a short experience with using a mitten to reach to distant objects, changes the infant perception of other goal directed actions, suggesting an important role for action experience on action observation. In support of this view, when babies perceived actions of others, they showed higher motor resonance for actions that were already present in their motor repertoire (e.g., crawling), compared to actions were not yet present in their repertoire (e.g., walking) (van Elk et al., 2008). Other studies also highlight the importance of visuo-motor experience and associative learning for the imitation of observed actions (for review, see Heyes, 2011).

If imitation is mediated by the mirror neuron system, then it might be possible to adjust imitative effects through sensorimotor learning. This is exactly what Heyes and colleagues tested in several experiments (Heyes et al., 2005; Catmur et al., 2008). They showed that humans make faster imitative gestures than comparable non-imitative gestures—an effect believed to be mediated by the mirror neuron system. However, they were able to change this advantage of imitative over non-imitative gestures through a sensorimotor training. In this training people were instructed to execute a particular action while observing a different action, thereby weakening existing imitative responses through interference. The finding that sensorimotor experience can cancel or even reverse automatic imitation was recently also corroborated by several other studies (Catmur et al., 2007; Press et al., 2007; Gillmeister et al., 2008), underlining the learned nature of imitative processes.

Although the ASL model can explain how infants learn to imitate through sensorimotor experience, the model lacks an explanation for the tongue protrusions found in neonates within 1 day after birth. Neonates that have only been born for a few hours lack the observational and action experience necessary for any imitative learning. Therefore, we propose to view such neonatal tongue protrusions—in line with Jones (2009)—not as genuine imitation, but as an innate tendency to explore the world instead. The ASL model can then still be used to explain the later development of genuine imitation in infants as being caused by sensorimotor experience^6^.

### 4.2. Implications for the enactivist theory of intersubjective understanding

Based on the studies reviewed in this paper, we conclude there is no strong evidence for innate and genuine neonate imitation. In fact, imitation may be learned and shaped through sensorimotor experience rather than being automatic and innate. A neonate's tongue protrusion can be explained as an innate tendency to explore the world, rather than being genuine imitation (Jones, 2009). This explanation, however, does not necessarily contradict the enactivist proposal that such tongue protrusions have a communicative or social function. Even if tongue protrusions turn out to be an a innate reflex, then this could still be a reflex that evolved biologically with a social function, because such neonatal gestures might stimulate the neonate's bonding with its parents, who likely adore such gestures.

If we assume that genuine imitation is learned through sensorimotor experience rather than being innate, then what are the implications for the enactivist theory in general and for the way it explains our intersubjective understanding? One implication would be that *nativist* enactivists are not warranted to claim that neonatal imitation supports the existence of intersubjective understanding in neonates. However, they could still use other studies to support the existence of infant intersubjectivity. For instance, Baron-Cohen (1997) describes two mechanisms that point to a basic intersubjective understanding in young infants. First, the eye-direction detector allows infants to recognize where other persons are looking and understand that a person is actually seeing something. Second, an intentionality detector allows infants to interpret bodily movement as goal-directed and intentional. One study showed that 18-month-old children could understand what another person intends to do and even finish the behavior if the observed person did not complete it (Baldwin and Baird, 2001). Other evidence on infant intersubjectivity shows that infants between 2 and 5 days old have a preference for looking at human faces (Farroni et al., 2002). Furthermore, 2–3 month old infants show awareness of their mother's emotional behavior by responding reciprocally (Murray and Trevarthen, 1985, 1986). The evidence described above, however, is based on studies that tested infants older than the ones used in the neonatal imitation experiments. Because of this time gap, infants already could have experienced interactions with other humans for at least a few days. Therefore one could argue that those findings can alternatively (and more parsimoniously) be explained as resulting from learning through social interaction. Because infants were not tested directly after birth, these findings cannot support an innate view as strongly as neonate imitation studies would do. In neonate imitation studies, neonates are sometimes observed within minutes after birth, which precludes the possibility of having experience with imitation. Therefore, if one wants to claim that innate processes are causally powerful then the studies used to support that claim will have to rule out that those processes are carved through learning.

The absence of neonate imitation evidence makes it more difficult for nativist enactivists to describe intersubjective understanding as an innate mechanism. It could still be the case, however, that these processes *are* present at birth, but then the nativist enactivist who uses neonate imitation studies will have to come up with new empirical evidence instead to support the claim that our basic intersubjective mechanisms are innate. Innateness, however, is not a necessary component of the enactivist theory in general. Empiricist enactivism, which proposes that the embodied processes underlying intersubjective understanding are learned rather than innate, is therefore not affected by the invalidity of neonate imitation. Nativist enactivists use the body schema as a mechanism to explain imitation and our understanding of others (Zahavi, 2001; Gallagher, 2005). The validity of that proposal is not necessarily threatened if genuine neonate imitation does not exist. We propose that mechanisms like the body schema and processes like imitation and social understanding are not innate, but need to be learned over time. The implication for enactivism would be that rather than being innate, the body schema is acquired through a process of exploration, sensorimotor experiences and learning from social interaction. Therefore, we claim that the available experimental evidence on neonate imitation only undermines the nativist enactivist view on intersubjective understanding, while the evidence does *not* contradict the *empiricist* enactivist views (Di Paolo and De Jaegher, 2012; Froese et al., 2012).

## 5. Conclusion

Altogether, the generality of genuine neonatal imitation is not supported convincingly by the available experimental evidence at this moment. Despite the findings of the tongue protrusion imitation, it cannot be concluded that neonate imitation is a general phenomenon. This conclusion provides a potential problem for the nativist enactivist proposal that neonates already have a basic and innate form of intersubjective understanding at birth. It would be important to address the contradictory findings in future theories regarding the innateness of social cognition and enactive understanding and to consider more parsimonious explanations of the tongue protrusion effect. Nonetheless, the outcome of the neonatal imitation debate does not pose a threat to enactivism in general, because other strands of evidence provide converging evidence for the importance of intersubjective processes in adult social cognition. The available evidence on neonatal imitation, however, calls for a more careful view on the innateness of such processes and suggests that this way of interacting needs to be learned over time.

### Conflict of interest statement

The authors declare that the research was conducted in the absence of any commercial or financial relationships that could be construed as a potential conflict of interest.

## Footnotes

^1^We realize that the word “understanding” has a strong cognitivist connotation, when combined with words like “intention,” but in our view the term understanding in itself can be used by both cognitivists and enactivists alike, because understanding can also be interpreted in a non-cognitivist way. For instance, Gallagher and Hutto (2008) published an article titled: “Understanding others through primary interaction and narrative practice.” Carpendale and Lewis (2010) define social understanding as the “everyday thinking necessary to engage in social interaction.” Because this definition could imply a cognitivist reading of social understanding and we aim to remain agnostic regarding the debate on the role of representations when it comes to explaining social interaction, we propose to define social understanding as “the skills necessary to engage in social interaction.” Social understanding from a cognitivist perspective would for instance involve skills like having mental representations about other people's intentions, while from an enactivist perspective it would for instance involve skills like an immediate perceptual understanding arising from a social interaction in which intentions are explicitly expressed in embodied actions Gallagher and Hutto (2008).

^2^We realize that many enactivist positions are more nuanced than the nativist-empiricist distinction suggests, but we still consider our distinction useful because it provides a conceptual tool for classifying different theories in their relative emphasis on learning or innate processes. That is, we argue that some enactivist explain social understanding in part by innate processes, while other enactivists deny the relevance of such processes because they claim that interactive or learning processes are sufficient. The distinction between nativist and empiricist enactivism therefore primarily serves an instrumental purpose in order to illustrate the differing enactivist views on the origin of social understanding. A similar empiricist-nativist distinction appears to be a fruitful way to classify other developmental debates, such as the origin of knowledge (Spelke, 1998), language (MacWhinney, 1999), or spatial and quantitative processing (Newcombe, 2002). We propose to use a similar distinction to clarify the present debate on the origin of social understanding. Disentangling theories based on their relative emphasis on learning or innate processes is especially relevant for discussing the evidence of neonatal imitation. That is, if neonatal imitation would exist, this provides strong evidence for the notion that basic forms of social interaction are already present at birth and do not have to be learned.

^3^Some enactivists, however, do not necessarilly view two qualitatively different forms of imitation (neonate vs. adult) as problematic (Froese and Leavens, 2014)

^4^This experiment by itself does in our view not provide evidence for the nativist enactivist claim that neonates are capable of intersubjective understanding, for all the dynamics between actor and observer are lost after the introduction of a delay between the modeled gesture and the neonate's response.

^5^Such a meta-analysis, however, was beyond the scope of the present paper.

^6^One shortcoming of all explanations described above, however, is that they all focus on individuals as units of analysis. This “methodological individualism” (Boden, 2006) is not only dominant in imitation research, but also in most areas of social neuroscience. Recently, a new model has been proposed (Froese et al., 2012) that explains imitation not only in terms of the individuals involved in the imitation, but takes the social interaction itself as a unit of analysis. This theory actually bypasses the nativist-enactivist discussion, because instead of using individual mechanisms (innate vs. learned), it explains imitation as emerging completely from the social interaction itself. Although this theory has been supported experimentally (Froese et al., 2012), it is not yet complemented by brain imaging studies because of the challenges associated with second-person perspective neuroscience. A potential venue of future research would therefore be to study the social interaction underlying imitation by using promising new second-person perspective techniques such as dual EEG (Dumas et al., 2010; Naeem et al., 2012).

## References

[B1] Abravanel E. DeYong N. G. (1991). Does object modeling elicit imitative-like gestures from young infants? J. Exp. Child Psychol. 52, 22–40 10.1016/0022-0965(91)90004-C 1890381

[B2] Abravanel E. Sigafoos A. D. (1984). Exploring the presence of imitation during early infancy. Child Dev. 55, 381–392 10.2307/1129950 6723442

[B3] Akins C. K. Zentall T. R. (1996). Imitative learning in male japanese quail (coturnix japonica) using the two-action method. J. Comp. Psychol. 110, 316 10.1037/0735-7036.110.3.316 8858851

[B4] Akins C. K. Zentall T. R. (1998). Imitation in japanese quail: the role of reinforcement of demonstrator responding. Psychon. Bull. Rev. 5, 694–697 10.3758/BF03208847

[B5] Anisfeld M. (1991). Neonatal imitation. Dev. Rev. 11, 60–97 10.1016/0273-2297(91)90003-7

[B6] Anisfeld M. (1996). Only tongue protrusion modeling is matched by neonates. Dev. Rev. 16, 149–161 10.1006/drev.1996.0006

[B7] Anisfeld M. Masters J. C. Jacobson S. W. Kagan J. (1979). Interpreting imitative responses in early infancy. Science 205, 214–215 10.1126/science.451593 451595

[B8] Baldwin D. A. Baird J. A. (2001). Discerning intentions in dynamic human action. Trends Cogn. Sci. 5, 171–178 10.1016/S1364-6613(00)01615-6 11287271

[B9] Bard K. A. (2007). Neonatal imitation in chimpanzees (pan troglodytes) tested with two paradigms. Anim. Cogn. 10, 233–242 10.1007/s10071-006-0062-3 17180698

[B10] Baron-Cohen S. (1997). Mindblindness: An Essay on Autism and Theory of Mind. Cambridge, MA: MIT press

[B11] Bjorklund D. F. (1987). A note on neonatal imitation. Dev. Rev. 7, 86–92 10.1016/0273-2297(87)90006-2

[B12] Boden M. (2006). Of islands and interactions. J. Conscious. Stud. 13, 53–63

[B13] Brass M. Bekkering H. Prinz W. (2001). Movement observation affects movement execution in a simple response task. Acta Psychol. 106, 3–22 10.1016/S0001-6918(00)00024-X 11256338

[B14] Butterworth G. (1992). Origins of self-perception in infancy. Psychol. Inq. 3, 103–111 10.1207/s15327965pli0302_1

[B15] Calvo-Merino B. Glaser D. E. Grèzes J. Passingham R. E. Haggard P. (2005). Action observation and acquired motor skills: an fmri study with expert dancers. Cereb. Cortex 15, 1243–1249 10.1093/cercor/bhi007 15616133

[B16] Calvo-Merino B. Grèzes J. Glaser D. E. Passingham R. E. Haggard P. (2006). Seeing or doing? influence of visual and motor familiarity in action observation. Curr. Biol. 16, 1905–1910 10.1016/j.cub.2006.07.065 17027486

[B17] Carpendale J. I. Lewis C. (2010). The development of social understanding, in The Handbook Life-Span Development, eds Lerner R. M. Overton W. F. (Hoboken, NJ: Wiley), 548–627

[B18] Carpenter M. Akhtar N. Tomasello M. (1998). Fourteen-through 18-month-old infants differentially imitate intentional and accidental actions. Infant Behav. Dev. 21, 315–330 10.1016/S0163-6383(98)90009-1

[B19] Carpenter M. Tomasello M. (1995). Joint attention and imitative learning in children, chimpanzees, and enculturated chimpanzees*. Soc. Dev. 4, 217–237 10.1111/j.1467-9507.1995.tb00063.x

[B20] Catmur C. Gillmeister H. Bird G. Liepelt R. Brass M. Heyes C. (2008). Through the looking glass: counter-mirror activation following incompatible sensorimotor learning. Eur. J. Neurosci. 28, 1208–1215 10.1111/j.1460-9568.2008.06419.x 18783371

[B21] Catmur C. Walsh V. Heyes C. (2007). Sensorimotor learning configures the human mirror system. Curr. Biol. 17, 1527–1531 10.1016/j.cub.2007.08.006 17716898

[B22] Catmur C. Walsh V. Heyes C. (2009). Associative sequence learning: the role of experience in the development of imitation and the mirror system. Philos. Trans. R. Soc. B Biol. Sci. 364, 2369–2380 10.1098/rstb.2009.0048 19620108 PMC2865072

[B23] Chemero A. (2009). Radical Embodied Cognitive Science. Cambridge, MA: MIT press

[B24] Cohen J. (1977). Statistical Power Analysis for the Behavioral Sciences. Hillsdale, NJ: Lawrence Erlbaum Associates, Inc

[B25] Custance D. Whiten A. Fredman T. (1999). Social learning of an artificial fruit task in capuchin monkeys (cebus apella). J. Comp. Psychol. 113, 13 10.1037/0735-7036.113.1.13

[B26] Custance D. M. Whiten A. Bard K. A. (1995). Can young chimpanzees (pan troglodytes) imitate arbitrary actions? hayes & hayes (1952) revisited. Behaviour 132, 837–859 10.1163/156853995X00036

[B27] de Bruin L. C. Kästner L. (2012). Dynamic embodied cognition. Phenomenol. Cogn. Sci. 11, 541–563 10.1007/s11097-011-9223-1

[B28] De Vignemont F. (2003). Ghost buster: the reality of one's own body. Theor. Hist. Sci. 7, 121–140

[B29] Di Paolo E. De Jaegher H. (2012). The interactive brain hypothesis. Front. Hum. Neurosci. 6:163 10.3389/fnhum.2012.00163 22701412 PMC3369190

[B30] Dumas G. Nadel J. Soussignan R. Martinerie J. Garnero L. (2010). Inter-brain synchronization during social interaction. PLoS ONE 5:e12166 10.1371/journal.pone.0012166 20808907 PMC2923151

[B31] Farroni T. Csibra G. Simion F. Johnson M. H. (2002). Eye contact detection in humans from birth. Proc. Natl. Acad. Sci. U.S.A. 99, 9602–9605 10.1073/pnas.152159999 12082186 PMC123187

[B32] Ferrari P. F. Visalberghi E. Paukner A. Fogassi L. Ruggiero A. Suomi S. J. (2006). Neonatal imitation in rhesus macaques. PLoS Biol. 4:e302 10.1371/journal.pbio.0040302 16953662 PMC1560174

[B33] Field T. M. Woodson R. Greenberg R. Cohen D. (1983). Discrimination and imitation of facial expressions by neonates. Annu. Prog. Child Psychiatry Child Dev. 16, 119–125 10.1016/S0163-6383(83)90316-8 7123230

[B34] Fontaine R. (1984). Imitative skills between birth and six months. Infant Behav. Dev. 7, 323–333 10.1016/S0163-6383(84)80047-8

[B35] Froese T. Leavens D. A. (2014). The direct perception hypothesis: perceiving the intention of another action hinders its precise imitation. Front. Psychol. 5:65 10.3389/fpsyg.2014.00065 24600413 PMC3927096

[B36] Froese T. Lenay C. Ikegami T. (2012). Imitation by social interaction? analysis of a minimal agent-based model of the correspondence problem. Front. Hum. Neurosci. 6:202 10.3389/fnhum.2012.00202 23060768 PMC3463947

[B37] Fuchs T. (2009). Embodied cognitive neuroscience and its consequences for psychiatry. Poiesis Prax. 6, 219–233 10.1007/s10202-008-0068-9

[B38] Fuchs T. De Jaegher H. (2009). Enactive intersubjectivity: participatory sense-making and mutual incorporation. Phenomenol. Cogn. Sci. 8, 465–486 10.1007/s11097-009-9136-4

[B39] Gallagher S. (2000). Philosophical conceptions of the self: implications for cognitive science. Trends Cogn. Sci. 4, 14–21 10.1016/S1364-6613(99)01417-5 10637618

[B40] Gallagher S. (2001). The practice of mind. theory, simulation or primary interaction? J. Conscious. Stud. 8, 5–7

[B41] Gallagher S. (2005). How the Body Shapes the Mind. Cambridge, MA: Cambridge University Press 10.1093/0199271941.001.0001

[B42] Gallagher S. (2008). Inference or interaction: social cognition without precursors. Philos. Exp. 11, 163–174 10.1080/13869790802239227

[B43] Gallagher S. (2011). Narrative competency and the massive hermeneutical background, in Education, Dialogue, and Hermeneutics, ed Fairfield P. (New York, NY: Continuum), 21–38

[B44] Gallagher S. Hutto D. (2008). Understanding others through primary interaction and narrative practice, in The Shared Mind: Perspectives on Intersubjectivity, eds Zlatev J. Racine T. Sinha C. Itkonen E. (Amsterdam: John Benjamins), 17–38 10.1075/celcr.12.04gal

[B45] Gallagher S. Meltzoff A. N. (1996). The earliest sense of self and others: Merleau-ponty and recent developmental studies. Philos. Psychol. 9, 211–233 10.1080/09515089608573181 PMC3845406 24307757

[B46] Gallese V. Keysers C. Rizzolatti G. (2004). A unifying view of the basis of social cognition. Trends Cogn. Sci. 8, 396–403 10.1016/j.tics.2004.07.002 15350240

[B47] Galloway J. C. Thelen E. (2004). Feet first: object exploration in young infants. Infant Behav. Dev. 27, 107–112 10.1016/j.infbeh.2003.06.001

[B48] Gershkoff-Stowe L. Thelen E. (2004). U-shaped changes in behavior: a dynamic systems perspective. J. Cogn. Dev. 5, 11–36 10.1207/s15327647jcd0501_219702398

[B49] Gillmeister H. Catmur C. Liepelt R. Brass M. Heyes C. (2008). Experience-based priming of body parts: a study of action imitation. Brain Res. 1217, 157–170 10.1016/j.brainres.2007.12.076 18502404

[B50] Goldman A. I. (2002). Simulation theory and mental concepts. Adv. Conscious. Res. 45, 1–20 10.1075/aicr.45.02gol

[B51] Gopnik A. Wellman H. M. (1994). The theory theory, in An Earlier Version of This Chapter was Presented at The Society for Research in Child Development Meeting, 1991 (Cambridge, MA: Cambridge University Press).

[B52] Gordon R. M. (1986). Folk psychology as simulation. Mind Lang. 1, 158–171 10.1111/j.1468-0017.1986.tb00324.x

[B53] Hayes L. A. Watson J. S. (1981). Neonatal imitation: fact or artifact? Dev. Psychol. 17, 655 10.1037/0012-1649.17.5.655

[B54] Heimann M. Nelson K. E. Schaller J. (1989). Neonatal imitation of tongue protrusion and mouth opening: methodological aspects and evidence of early individual differences. Scand. J. Psychol. 30, 90–101 10.1111/j.1467-9450.1989.tb01072.x 2772571

[B55] Heyes C. (2001). Causes and consequences of imitation. Trends Cogn. Sci. 5, 253–261 10.1016/S1364-6613(00)01661-2 11390296

[B56] Heyes C. (2002). Transformational and associative theories of imitation, in Imitation in Animals and Artifacts, eds Dautenhahn K. Nehaniv C. L. (Cambridge, MA: MIT Press), 501–523

[B57] Heyes C. (2011). Automatic imitation. Psychol. Bull. 137, 463 10.1037/a0022288 21280938

[B58] Heyes C. Bird G. Johnson H. Haggard P. (2005). Experience modulates automatic imitation. Cogn. Brain Res. 22, 233–240 10.1016/j.cogbrainres.2004.09.009 15653296

[B59] Heyes C. M. Ray E. D. (2000). What is the significance of imitation in animals? Adv. Stud. Behav. 29, 215–245 5377343

[B60] Hox J. (2010). Multilevel Analysis: Techniques and Applications. New York, NY: Routledge

[B61] Hox J. J. (2002). Multilevel Analysis: Techniques and Applications. New York, NY: Psychology Press

[B62] Jacobson S. W. (1979). Matching behavior in the young infant. Child Dev. 50, 425–430 10.2307/1129418 487882

[B63a] Jones S. S. (1996). Imitation or exploration? young infants' matching of adults' oral gestures. experiment 1. Child Dev. 67, 1952–1969 10.2307/1131603 9022224

[B63b] Jones S. S. (1996). Imitation or exploration? young infants' matching of adults' oral gestures. experiment 3. Child Dev. 67, 1952–1969 10.2307/1131603 9022224

[B65] Jones S. S. (2009). The development of imitation in infancy. Philos. Trans. R. Soc. B Biol. Sci. 364, 2325–2335 10.1098/rstb.2009.0045 19620104 PMC2865075

[B66] Kilner J. Paulignan Y. Blakemore S. (2003). An interference effect of observed biological movement on action. Curr. Biol. 13, 522–525 10.1016/S0960-9822(03)00165-9 12646137

[B67] Koepke J. E. Hamm M. Legerstee M. Russell M. (1983). Neonatal imitation: two failures to replicate. Infant Behav. Dev. 6, 97–102 10.1016/S0163-6383(83)80012-5

[B68] Kugiumutzakis J. (1985). The Origin, Development, and Function of The Early Infant Imitation. Ph.D. thesis, Uppsala University

[B69] Leslie A. M. (1987). Pretense and representation: the origins of theory of mind. Psychol. Rev. 94, 412 10.1037/0033-295X.94.4.412

[B70] Lewis M. Sullivan M. W. (1985). Imitation in the first six months of life, Merrill. Palmer. Q. 31, 315–333 6668235

[B71] Longo M. R. Kosobud A. Bertenthal B. I. (2008). Automatic imitation of biomechanically possible and impossible actions: effects of priming movements versus goals. J. Exp. Psychol. Hum. Percept. Perform. 34, 489 10.1037/0096-1523.34.2.489 18377184

[B72] MacWhinney B. (1999). The Emergence of Language. Mahwah, NJ: Taylor & Francis

[B73] McDougall (1926). An Introduction to Social Psychology. London: Dover Publications

[B74] McKenzie B. Over R. (1983). Young infants fail to imitate facial and manual gestures. Infant Behav. Dev. 6, 85–95 10.1016/S0163-6383(83)80011-3

[B75] Meltzoff A. N. (1995). Understanding the intentions of others: re-enactment of intended acts by 18-month-old children. Dev. Psychol. 31, 838 10.1037/0012-1649.31.5.838 25147406 PMC4137788

[B76] Meltzoff A. N. (2002). Imitation as a mechanism of social cognition: origins of empathy, theory of mind, and the representation of action, in Blackwell Handbook of Childhood Cognitive Development, eds Whitbourne S. K. Sliwinski M. (West Sussex: Wiley), 6–25 10.1002/9780470996652.ch1

[B77] Meltzoff A. N. Moore K. M. (1994). Imitation, memory, and the representation of persons. Infant Behav. Dev. 17, 83–99 10.1016/0163-6383(94)90024-8 25147416 PMC4137868

[B78] Meltzoff A. N. Moore M. K. (1977). Imitation of facial and manual gestures by human neonates. Science 198, 75–78 10.1126/science.198.4312.75 17741897

[B79] Meltzoff A. N. Moore M. K. (1983a). Newborn infants imitate adult facial gestures. Child Dev. 54, 702–709 10.2307/1130058 6851717

[B80] Meltzoff A. N. Moore M. K. (1983b). The origins of imitation in infancy: paradigm, phenomena, and theories. Adv. Infancy Res. 2, 265–301

[B81] Meltzoff A. N. Moore M. K. (1989). Imitation in newborn infants: exploring the range of gestures imitated and the underlying mechanisms. Dev. Psychol. 25, 954 10.1037/0012-1649.25.6.954 25147405 PMC4137867

[B82] Murray L. Trevarthen C. (1985). Emotional regulation of interactions between two-month-olds and their mothers, in Social Perception in Infants, eds Field T. M. Fox N. A. (Norwood, NJ: Ablex Publishing Corporation), 177–197

[B83] Murray L. Trevarthen C. (1986). The infant's role in mother–infant communications. J. Child Lang. 13, 15–29 10.1017/S0305000900000271 3949895

[B84] Myowa-Yamakoshi M. (2006). How and when do chimpanzees acquire the ability to imitate?, in Cognitive Development in Chimpanzees, eds Matsuzawa T. Tomonaga M. Tanaka M. (Tokyo: Springer), 214–232 10.1007/4-431-30248-4_14

[B85] Nadel J. E. Butterworth G. E. (1999). Imitation in Infancy. Cambridge, MA: Cambridge University Press

[B86] Naeem M. Prasad G. Watson D. R. Kelso J. (2012). Electrophysiological signatures of intentional social coordination in the 10–12hz range. Neuroimage 59, 1795–1803 10.1016/j.neuroimage.2011.08.010 21871572

[B87] Neuberger J. Helmut Merz J. Selg H. (1983). Imitation in newborn infants: a controversial finding. Fur Dev. Psychol. Pedag. Psychol. 15, 267–276

[B88] Newcombe N. S. (2002). The nativist-empiricist controversy in the context of recent research on spatial and quantitative development. Psychol. Sci. 13, 395–401 10.1111/1467-9280.00471 12219804

[B89] Piaget J. (1946). Development of The Concept of Time in Children. Paris: Presses University of France

[B90] Press C. Bird G. Flach R. Heyes C. (2005). Robotic movement elicits automatic imitation. Cogn. Brain Res. 25, 632–640 10.1016/j.cogbrainres.2005.08.020 16344220

[B91] Press C. Gillmeister H. Heyes C. (2007). Sensorimotor experience enhances automatic imitation of robotic action. Proc. R. Soc. B Biol. Sci. 274, 2509–2514 10.1098/rspb.2007.0774 17698489 PMC2275887

[B92] Ray E. Heyes C. (2011). Imitation in infancy: the wealth of the stimulus. Dev. Sci. 14, 92–105 10.1111/j.1467-7687.2010.00961.x 21159091

[B93] Rizzolatti G. Fogassi L. Gallese V. (2001). Neurophysiological mechanisms underlying the understanding and imitation of action. Nat. Rev. Neurosci. 2, 661–670 10.1038/35090060 11533734

[B94] Schaal S. (1999). Is imitation learning the route to humanoid robots? Trends Cogn. Sci. 3, 233–242 10.1016/S1364-6613(99)01327-3 10354577

[B95] Schultz W. Dickinson A. (2000). Neuronal coding of prediction errors. Annu. Rev. Neurosci. 23, 473–500 10.1146/annurev.neuro.23.1.473 10845072

[B96] Sommerville J. A. Woodward A. L. Needham A. (2005). Action experience alters 3-month-old infants' perception of others' actions. Cognition 96, B1–B11 10.1016/j.cognition.2004.07.004 15833301 PMC3908452

[B97] Spaulding S. (2010). Embodied cognition and mindreading. Mind Lang. 25, 119–140 10.1111/j.1468-0017.2009.01383.x

[B98] Spelke E. S. (1998). Nativism, empiricism, and the origins of knowledge. Infant Behav. Dev. 21, 181–200 10.1016/S0163-6383(98)90002-9

[B99] Stern W. Barwell A. T. (1924). Psychology of Early Childhood: Up to the Sixth Year of Age (rev. and enlarged). New York, NY: Henry Holt and Co

[B100] Stevens J. (2009). Applied Multivariate Statistics for The Social Sciences. New York, NY: Taylor & Francis US

[B101] Tabachnick B. G. Fidell L. S. Osterlind S. J. (2001). Using Multivariate Statistics. Needham Heights, MA: Allyn & Bacon US

[B102] Takahashi Y. Tamura Y. Asada M. Negrello M. (2010). Emulation and behavior understanding through shared values. Robot. Auton. Syst. 58, 855–865 10.1016/j.robot.2010.03.006

[B103] Tomasello M. (1996). Do apes ape?, in Social Learning in Animals: The Roots of Culture, eds Heyes C. M. Galef B. G. (New York, NY: Academic Press).

[B104] van Elk M. van Schie H. T. Hunnius S. Vesper C. Bekkering H. (2008). You'll never crawl alone: neurophysiological evidence for experience-dependent motor resonance in infancy. Neuroimage 43, 808–814 10.1016/j.neuroimage.2008.07.057 18760368

[B105] van Schie H. T. van Waterschoot B. M. Bekkering H. (2008). Understanding action beyond imitation: reversed compatibility effects of action observation in imitation and joint action. J. Exp. Psychol. Hum. Percept. Perform. 34, 1493 10.1037/a0011750 19045988

[B106] Varga S. Gallagher S. (2012). Critical social philosophy, honneth and the role of primary intersubjectivity. Eur. J. Soc. Theor. 15, 243–260 10.1177/1368431011423606

[B107] Zahavi D. (2001). Beyond empathy. phenomenological approaches to intersubjectivity. J. Conscious. Stud. 8, 5–7

[B108] Zmyj N. Jank J. Schütz-Bosbach S. Daum M. M. (2011). Detection of visual–tactile contingency in the first year after birth. Cognition 120, 82–89 10.1016/j.cognition.2011.03.001 21458785

